# Measuring the effect of residual stress on the machined subsurface of Inconel 718 by nanoindentation

**DOI:** 10.1371/journal.pone.0245391

**Published:** 2021-01-14

**Authors:** Ling Chen, Qirui Du, Miao Yu, Xin Guo, Wu Zhao

**Affiliations:** 1 School of Mechanical Engineering, Sichuan University, Chengdu, China; 2 Innovation Method and Creative Design Key Laboratory of Sichuan Province, Chengdu, China; China University of Mining and Technology, CHINA

## Abstract

Inconel 718 alloy is widely used in aero-engines and high-temperature environments. However, residual stress caused by processing and molding leads to an uneven distribution of internal pressure, which reduces the reliability of service process. Therefore, numerical simulation of the nanoindentation process was applied to evaluate the effect of residual stress on the machined subsurface of Inconel 718. A gradient material model of Inconel 718 was established in ABAQUS finite element software. Mechanical properties based on nanoindentation testing showed an influence of residual stress in combination with indenter geometry. The orthogonal experimental results show that under diverse residual stress states, the indenter’s geometry can affect the pile-up of the material surface after nanoindentation and significantly influence the test results. With increases in piling-up, the error caused by residual stress on the characterization of the mechanical properties of the hardened layer increases. Through the establishment of a numerical model, the influence of residual stress can be predicted within nanoindentation depths of 300 nm.

## Introduction

**Fig 1:**
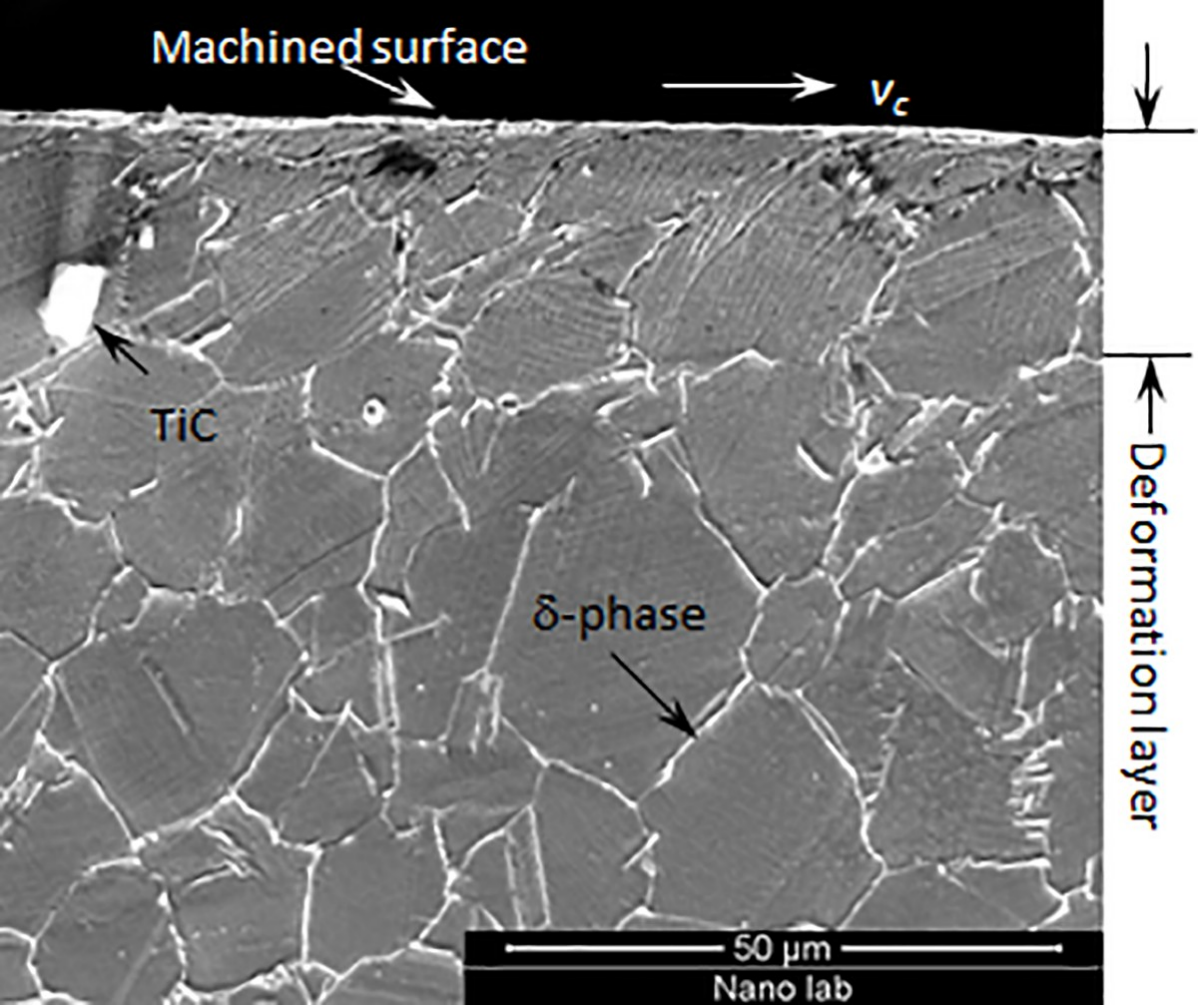
Typical subsurface deformation layer after processing of Inconel 718.

Inconel 718 is a nickel-chromium alloy that is essential in the aviation industry and is widely applied in aircraft engines and turbine motors. The surface quality of machined parts plays an essential role in the reliability of components. In the process of machining, the subsurface of machined parts often deforms seriously, and the microstructure may change in the small-scale subsurface layer. Fig 1 shows a typical subsurface layer deformed by machining, in which the grain structure is significantly altered due to the cutting force and thermal load. Particular residual stress is generated, which affects the mechanical properties of the material. The measurement and evaluation of residual stress are key to studying properties of thin films [1, 2].

In recent decades, nanoindentation techniques have been extensively developed and have become an important way to study the micro-nano mechanical properties of materials. Nanoindentation can measure the mechanical properties (e.g. nanohardness and modulus) of a material surface based on a microscale load-depth curve [3]. Dean et al. [4] measured the residual stress on the surface of a material through nanoindentation and proved that small changes in stress could can the detection results. Xu and Li [5] used finite element simulations to determine that the hardness (*H)* obtained by nanoindentation decreases slightly with increases in residual (tensile) stress. At the same time, the accuracy of a nanoindentation experiment determines the precision of residual stress prediction and evaluation, with accurate measurement of the contact area being key. Zambaldi et al. [6] discovered pile-up during nanoindentation simulation of cylinder specimens and in experimental testing of the γ-TiAl film [7]. When pile-up occurs, traditional methods are prone to measurement error in the contact area. Finite element simulation of nanoindentation experiments can provide a solution. Through the finite element analysis method, changes in a material’s surface can be predicted. FEM is widely used in the analysis of mechanical properties [8–10]. Additionally, many parameters can significantly influence the reliability of nanoindentation measurements, such as roughness tolerance of specimens or geometric deviations caused by manufacturing tolerance of the indenter tip [11–13]. Doerner and Nix pointed out that indenter tips are significantly blunter than the ideal pyramid shapes used in nanoindentation experiments due to the tip radius [14]. Bucaille and Wang studied the influence of the indenter angle on indentation behaviour through the FEM and confirmed that changes in the indenter angle significantly influence measurement results [15, 16].

The influence of indenter geometry on nanoindentation has been verified in many studies, which cannot be ignored when studying residual stress on the mechanical properties of materials. However, due to the difficulty of measuring the contact depth after nanoindentation, these effects are rarely considered. At the same time, the mechanical properties are not uniformly distributed inside due to the surface working hardening; therefore, it is difficult to quantify internal structural parameters via experiments. This paper uses ABAQUS software and numerical methods to establish a gradient model (FGM) of Inconel 718 alloy. The model is used to simulate increases in top hardness strength and decreases in internal mechanical properties due to work hardening [17]. Orthogonal methods and numerical regression were used to characterize the effects of residual stress, and the influence of indenter geometry on the mechanical properties was analyzed comprehensively. Finally, a multifactorial predictive model of residual stress was established, and the influences of residual stress within nanoindentation depths of 300 nm were predicted.

## Theory of measuring subsurface residual stress by nanoindentation

**Fig 2:**
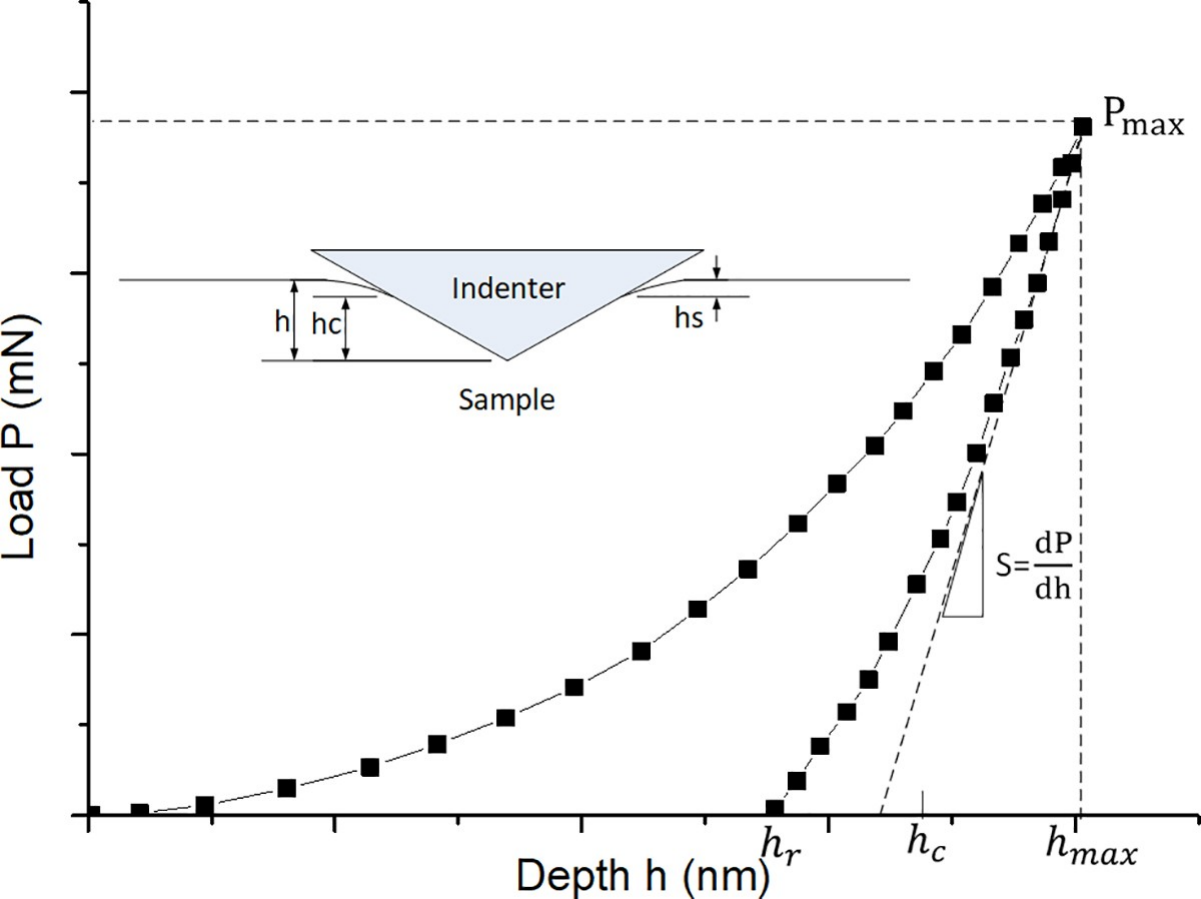
Typical load-depth curve from nanoindentation testing of a plastic material.

Nanoindentation is a type of indentation test that uses very low loads. The nanoindentation device is a kind of high-precision instrument that continuously records loads and displacement. A nanoindentation test starts with loading followed by unloading. A typical load-displacement curve obtained during the process is shown in Fig 2.

Several parameters can be determined from the load-displacement curve, such as the maximum indentation depth of the indenter (*h*_max_), maximum indentation load (*P*_max_), indenter-specimen contact depth (*h*_*c*_) and contact rigidity under the maximum indentation load (*S*). Based on these parameters, the mechanical properties like hardness (*H*_*IT*_) and Young’s modulus (*E*_*IT*_) of Inconel 718 alloy can be deduced [3]. The formula for calculating *H*_*IT*_ is:

$$H_{IT}=P_{max}/A_{p}(h_{c})$$ (1)

where *A*_*p*_(*h*_*c*_) is the contact area at the contact depth (*h*_*c*_). The Young’s modulus (*E*_*IT*_) is derived from the following formula:

$$E_{IT}=\frac{1-\nu_{S}^{2}}{1/E_{r}-(1-\nu_{i}^{2})/E_{i}}$$ (2)

where *E*_*IT*_ and *v*_*s*_ are the Young’s modulus and Poisson’s ratio of the specimen, respectively, while *E*_*i*_ and *v*_*i*_ are those of the indenter. For a diamond indenter, the values are *E*_*i*_ = 1140 GPa and *v*_*i*_ = 0.07, respectively. The modulus reduction (*E*_*r*_) of the elastic deformation of the indenter is defined as follows:

$$E_{r}={\left(\frac{\pi }{4}\right)}^{1/2}{\left(\frac{1}{A_{p}(h_{c})}\right)}^{1/2}{\left(\frac{dp}{dh}\right)}_{unloading}$$ (3)

Where (*dp*/*dh*)_unloading_ is experimentally measured rigidity at the upper part of the unloading curve. The contact area (*A*_*p*_) is determined by the area function *A*_*p*_(*h*_*c*_) and the indenter-specimen contact depth (*h*_*c*_) is determined as:

$$h_{c}=h_{max}-\varepsilon P_{max}/{\left(\frac{dp}{dh}\right)}_{unloading}$$ (4)

where *ε* is a geometric constant. For the Berkovich indenter used in this study, *ε* = 0.75.

For elastic solids, the stress-strain curve under a uniaxial tensile force is:

$$\varepsilon =\left\{ \begin{array}{c} \frac{\sigma }{E},\;\sigma <\sigma_{y}\\ \frac{\sigma_{y}}{E}{\left(\frac{\sigma }{\sigma_{y}}\right)}^{\frac{1}{n}},others \end{array}\right.$$ (5)

where *σ*_*y*_ is the initial yield stress, *n* is the working hardening index, and *E* is the Young’s modulus of the material. For most metals, *n* ranges between 0.1 and 0.5 [18]. In the present study, the influences of the hardening index on the experiment were ignored and *n* = 0. Besides, the surface roughness can reach a level far less than 300 nm using reasonable technology. Also, the influence of surface roughness can be ignored.

The models of Suresh and Giannakopoulos are most used to measure residual stress by nanoindentation. Tiwari et al. improved the formula and obtained a calculation formula that considers the change in the indenter angle [19]:

$$\sigma_{H}=\frac{H(1-\frac{A_{0}}{A})}{\sin\;\theta }$$ (6)

where a geometric factor, sin*θ*, is introduced, since the component of residual compressive stress that facilitates contact between the indenter and substrate acts in the direction normal to the inclined face of the indenter. *A*_0_ and *A* are obtained from the indentation stress-free and stressed samples, respectively.

According to Eqs (1) and (2), the precision of *A*_*p*_(*h*_*c*_) determines the precision of the calculated rigidity and Young’s modulus. Fischer-Cripps provided geometric equations of these indenters [20]. In the area function of an ideal indenter, the indenter is supposed to have a perfect geometric shape. In other words, the contact area is:

$$A_{p}=C_{0}{h_{c}}^{2}$$ (7)

where *C*_0_ is a fixed constant, and for an ideal Berkovich indenter, *C*_0_ = 24.56. In practical use, the indenter will generate a tip radius due to processing precision limitations and blunting [21]. Some researchers have proposed formulas to correct the projection contact areas and contact depths of Berkovich, conical and spherical indenters. The following formula can calculate the project area of a two-dimensional indenter [22]:

$$A_{p}=\left\{ \begin{array}{l} \pi h_{c}(2R-h_{c})\;for\;h_{c}\leq h-d\\ 24.5{\left(h_{c}+d\right)}^{2}\;for\;h_{c}>h-d \end{array}\right.$$ (8)

where the formulas for calculating *h* and *d* are:

$$h=\frac{R}{\sin\;\theta }-R\;\sin\;\theta$$

$$d=\frac{R}{\sin\;\theta }-R$$ (9)

In Eq (9), *R* is the tip radius. Shih viewed the Berkovich indenter as a trilateral pyramid with a total included angle of 142.3° [23]. The taper angle of an equivalent ideal indenter is θ = 70.3°. In this study, the projection contact area was calculated according to the two-dimensional method of Eq (8).

## FEM modeling and experiments

**Fig 3:**
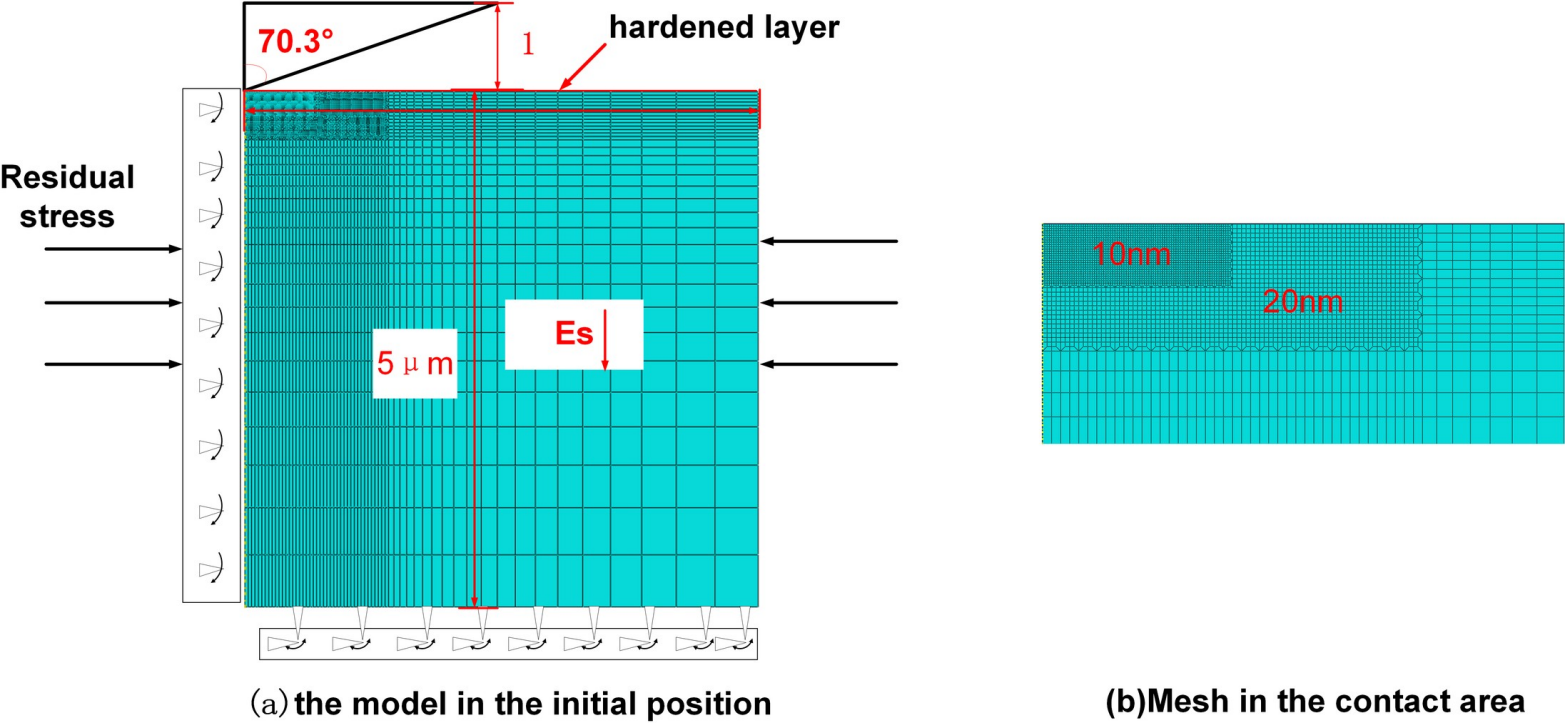
Components and initial elemental configuration of the finite element model. (a) the model in the initial position (b) mesh in the contact area.

It is hypothesized that Inconel 718 is a single-face material with a simple structure, and gradient materials were used as specimens. The elasticity decreases linearly along the vertical direction. Meanwhile, the material follows the Von-mises yield criterion, the elastic modulus of the upper hardening layer is *E*_*S*_ = 150 GPa and the gradient decreases to 140 GPa from the upper to lower positions. The yield stress is *σ*_*y*_ *=* 1.3 GPa and the Poisson’s ratio is *v*_*s*_ *=* 0.343 (Fig 3A).

In ABAQUS, uniaxial stress-strain data were input to simulate a nanoindentation experiment with an Inconel 718 gradient material with residual stress. The indenter and specimen were viewed as rotators for the finite element analysis, and an axisymmetric model was applied. This avoids the difficulty in simulating real pyramid indenters when there is a tip radius. Deformation of the indenter was neglected, and the indenter was viewed as a rigid body since its elastic modulus is far higher than that of the specimen. The conical half-angle and tip radius of a standard Berkovich indenter are 70.3° and 100 nm, respectively [24].

A specimen model was constructed using 11,038 four-node axial symmetric integral units (CAX4R element type). Fig 3A describes the model components in the initial state. To describe the gradients in the deformation and mechanical properties of specimens under indentation with sufficient accuracy, the grids close to the indenter were made ultrafine (10 nm). Grids further away from the indenter were gradually coarsened (Fig 3B).

The nanoindentation process is divided into loading and unloading stages. Displacement control was applied in simulation analysis. In the loading stage, vertical indentation was performed to a depth of 300 nm. In the unloading stage, the indenter was lifted up by 50 nm to separate it from the specimen. The contact constraints of the model were determined by the primary and secondary surfaces. The indenter was the primary surface, and the specimen was the secondary surface. The boundary conditions applied along the central line and at the bottom of the specimen are shown in Fig 3A.

To study the influences of the residual stress of nanoindentation on the mechanical properties of the materials, uniaxial stresses in the horizontal direction were set at four different levels from compressive stress to tensile stress, namely, 100, 150, 200, and 300 MPa, with the control group set as 0.

## Results and discussion

### Model verification

**Fig 4:**
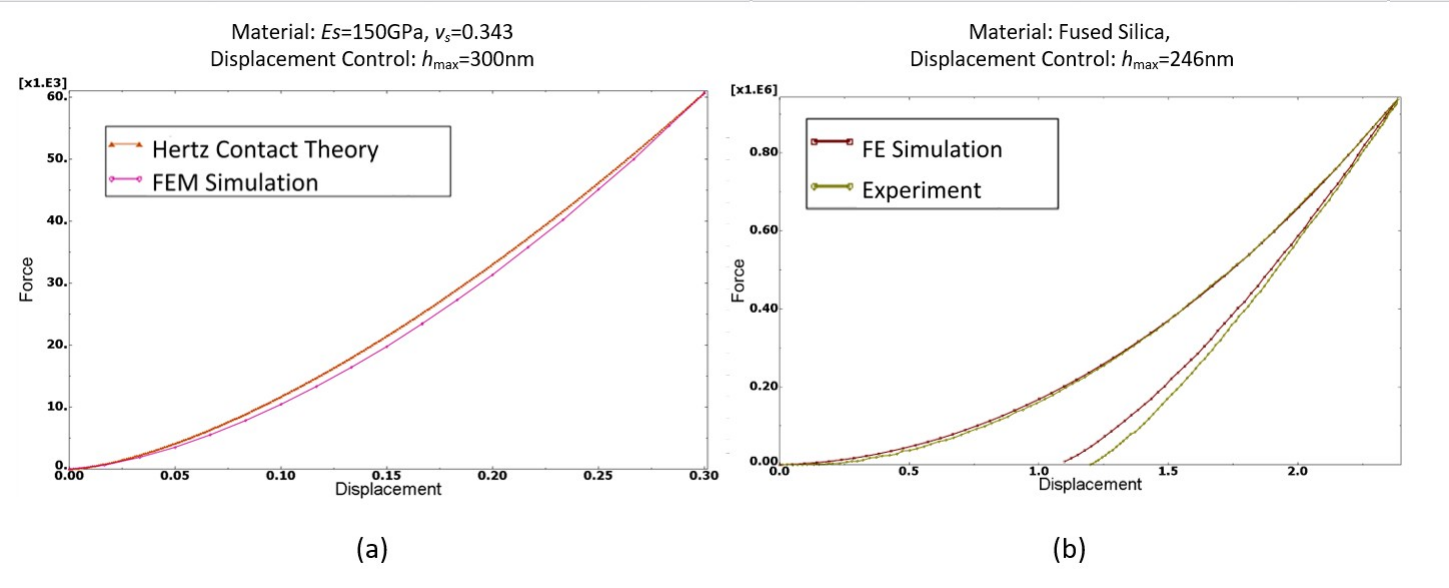
(a) Comparison of simulation results with Hertz contact theory and (b) simulated load-displacement curve and experimental curve for fused silica.

The FEM model was verified in two steps. Firstly, the elastic behaviors were compared with the Hertz contact theory. Secondly, elastoplastic behaviors were compared with experimental data. The elastic indentation was performed between the rigid sphere and semi-space of the Inconel 718 specimen and compared with the Hertz contact theory of loads. In the FEM simulation, a 300 nm indentation was applied to the spherical indenter with a radius of 3 μm. The results of the FEM model were highly consistent with Hertz theory (Fig 4A). Similarly, load-displacement curves gained from the simulation and experiment with fused silica were also highly consistent (Fig 4B) [25].

### Effects of residual stress

**Fig 5:**
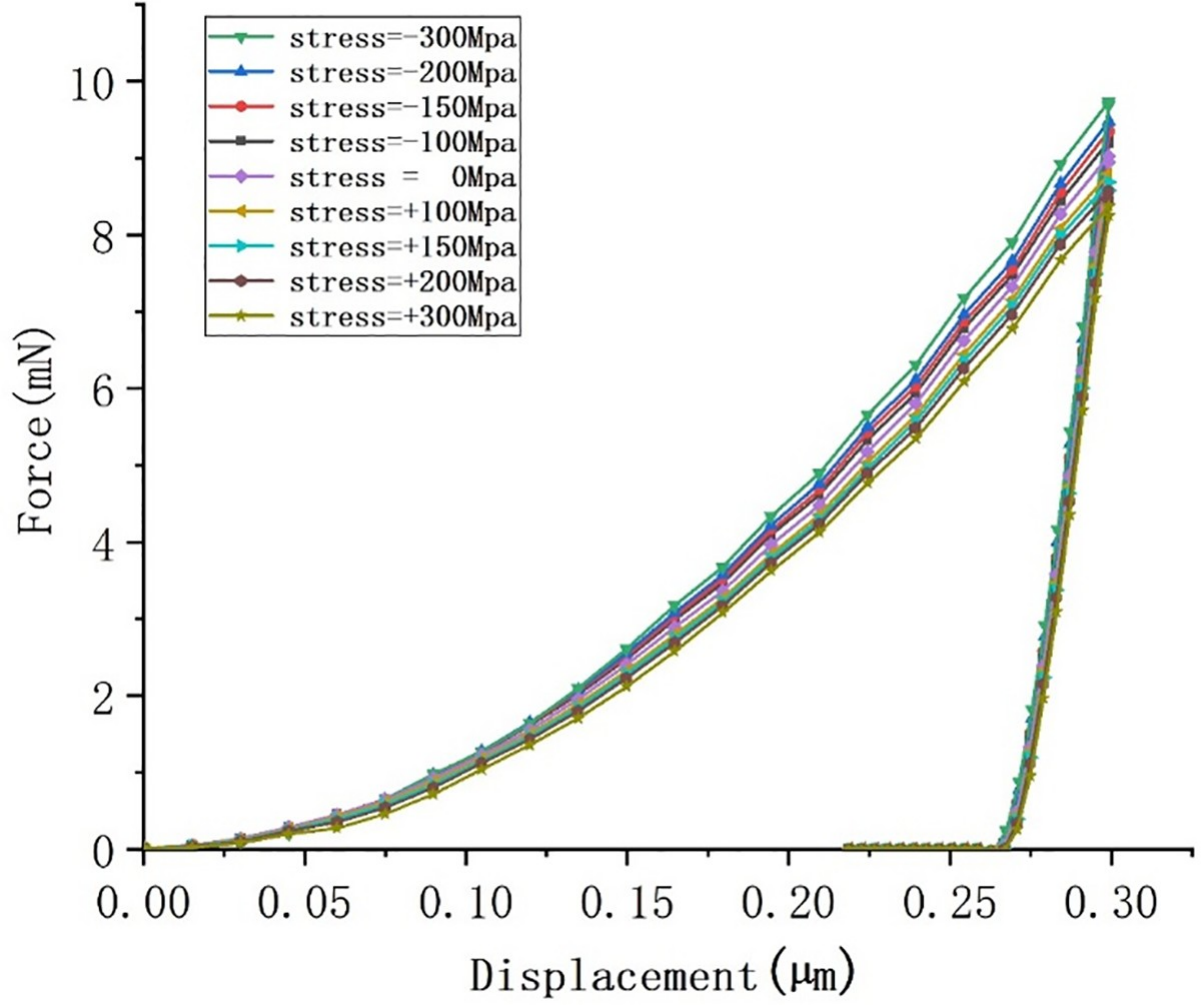
Load-displacement curves under different residual stresses.

Nine groups of horizontal uniaxial residual stresses were simulated, and the influences of residual stress were studied. Fig 5 shows a load-displacement curve for an indenter with different angles and a maximum depth of 300 nm. At a compressive stress of 300 MPa, the highest load was required to achieve the fixed nanoindentation depth. When the compressive stress was changed to tensile stress, the loads decreased gradually and the unloading curve moved rightward, indicating that the desired unloading displacement decreased. The results show that the nanoindentation load is positively related to residual compressive stress.

**Fig 6:**
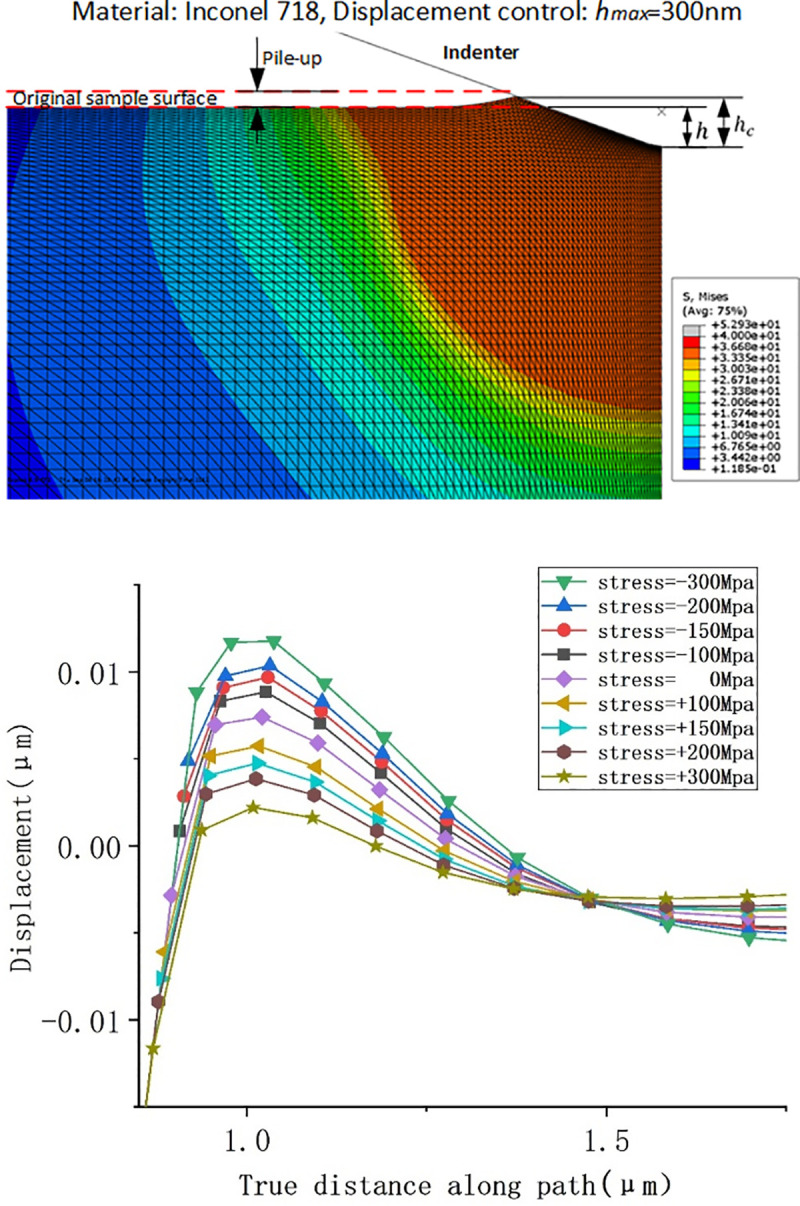
(a) Simulation of surface piling-up after a Berkovich indenter compressed Inconel 718 alloy and (b) pile-up heights at different stresses.

The contact depth between the indenter and specimen was calculated by the numerical method and FEM. In the numerical method, the maximum depth (*h*_max_) and rigidity ((*dp*/*dh*)_unloading_) were gained from each load-displacement curve of the FEM simulation. Eq (4) is based on Hertzian contact theory [26], which does not consider surface piling-up, resulting in a decrease in contact height and contact area, and increases in hardness and Young's modulus. Therefore, the FEM simulation results are more in line with the natural material’s characteristics, as shown in Fig 6A. Similarly, Fig 6B shows the pile-up height under different residual stresses. The height is 11.8 nm under the residual stress of −300 MPa, and the pile-up height decreases to 2.2 nm at 300 MPa. It can be indicated that the pile-up height is proportional to compressive residual stress. Significantly, residual stress influences the pile-up height on the surface state of materials during nanoindentation experiments.

**Fig 7:**
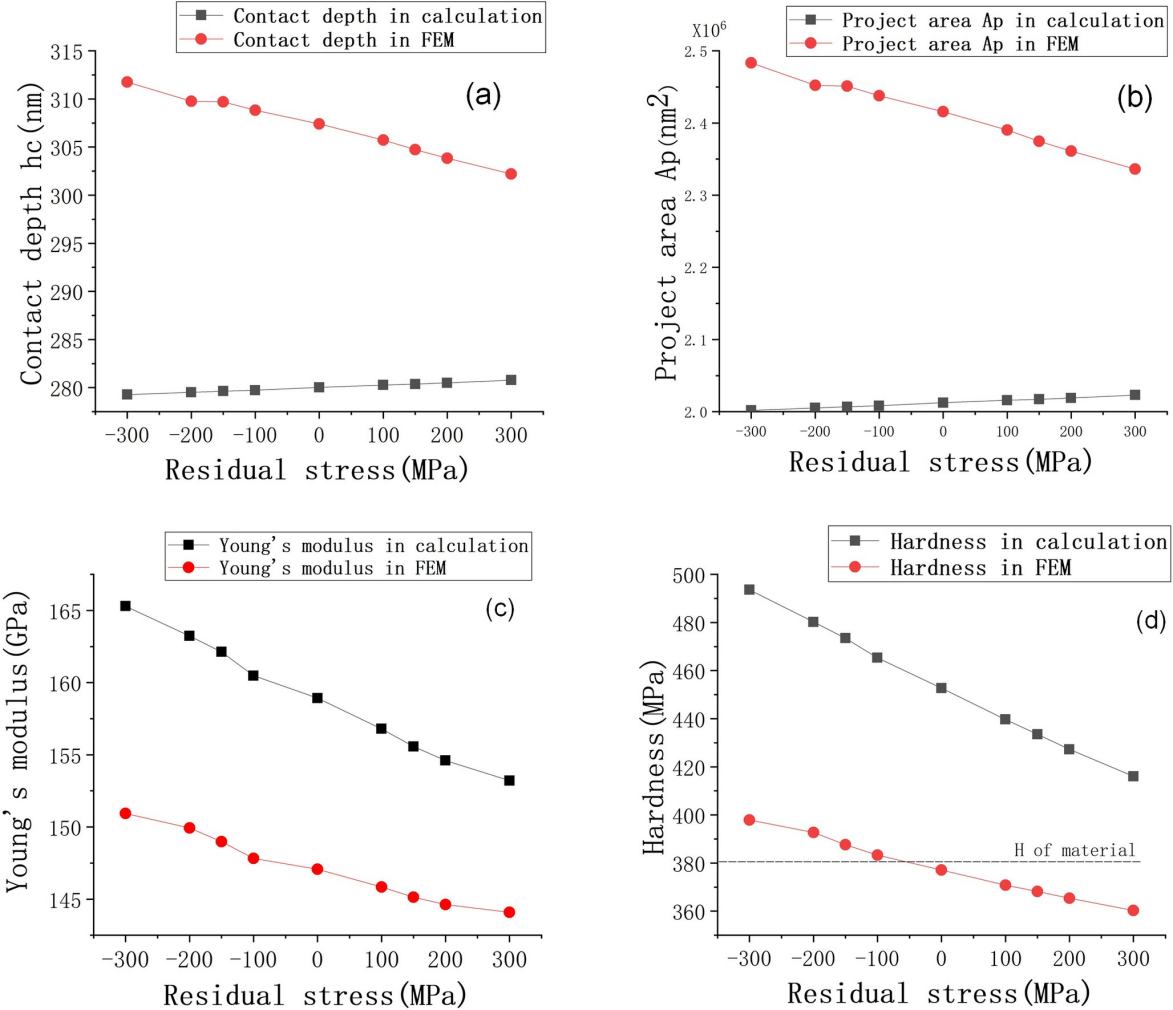
Influences of residual stress on (a) contact depth, (b) projected contact areas, (c) Young’s modulus and (d) hardness according to numerical and FEM methods.

By comparing the FEM and numerical calculation methods, the influence of material elastoplasticity on nanoindentation experimental results was obtained, as shown in Fig 7. The values of the mechanical properties estimated by the numerical calculation method are greater than those obtained by FEM. At −300 MPa, the difference in the hardness results of the two methods is 95.7 MPa, and that of Young's modulus is 14.4 GPa. Fig 7C and 7D shows the slope of the curve obtained by the two methods is different. As the residual stress changes from compressive stress to tensile stress, the difference between them will decrease. It is caused by the different contact depth, seen in Fig 7A. Therefore, for Inconel 718, the numerical calculation method does not consider material deformation due to pile-up, resulting in a large error in the calculations. The mechanical properties obtained are higher than the actual values, and the error gradually reduces with increases in residual tensile stress.

For a gradient material model, Young’s modulus close to the material surface is 150 GPa and it decreases linearly from top to bottom. According to the analysis of the nanoindentation results, the mechanical properties of a specimen surface can influence the final experimental results to a large extent, resulting in errors in performance measurement of overall materials. The numerical calculations and finite element simulation demonstrate linear reductions in Young’s modulus and hardness with increases in residual stress. The measured hardness decreases by 37 MPa, and Young’s modulus decreases by about 6.6 GPa as the residual stress changes from −300 MPa to 300 MPa. This indicates that residual stress can significantly influence the measured mechanical properties of materials. Moreover, the residual stress of materials can be predicted from Young’s modulus and hardness variations.

### Combined influences of three factors

In nanoindentation tests, the geometric shape of the indenter influences the accuracy of the measurements. Ideal indenters are not realistic since there is always a tip radius due to machining limitations and blunting during usage. The tip radius may become a critical issue due to the small scale of the indentations, especially for shallow indentations. Similarly, the cone half-angle of a standard Berkovich indenter is 70.3°, and the measurements vary according to the angle of the selected indenter. A three-factor and the five-level standard orthogonal test was designed. The influences of indenter angle, tip radius, and residual stress on nanoindentation tests were discussed. The factor-level table is shown in Table 1.

**Table 1:** Factor-level table.

Factor	Levels				
	1	2	3	4	5
Indenter angle θ (°)	55	60	65	70.3	75
Tip radius *r* (nm)	100	500	1000	1500	2000
Residual stress *σ* (MPa)	–300	–150	0	150	300

Table 2 shows the FEM simulation results of 25 groups of orthogonal experiments. The range analysis measurement method was applied for determining the influences of indenter angle, tip radius, and residual stress on the measured hardness and Young’s modulus. A higher range implies greater influences of relevant experimental parameters on the results. *Range* refers to the difference between the maximum and minimum average effect. The formula for calculating the range is:

$$R_{j}=Max(\bar{K_{j1}},\bar{K_{j2}},\ldots ,\bar{K_{jm}})-Min(\bar{K_{j1}},\bar{K_{j2}},\ldots ,\bar{K_{jm}})$$ (10)

where *R*_*j*_ is the amplitude of variation in the experimental parameters, with horizontal fluctuation in the factors in column *j*, and $\bar{K_{jm}}$ is the mean of test index sum corresponding to *m* level of factor *j*. The ranges of measured hardness and Young’s modulus were then calculated (Table 3).

**Table 2:** Simulation results of hardness and Young’s modulus.

Orthogonal experimental design					
No.	Indenter angle θ (°)	Tip radius *r* (nm)	Residual stress *σ* (MPa)	EIT (FEM)	HIT (FEM)
1	55	100	–300	142.55	386.17
2	60	500	–300	150.84	382.52
3	65	1000	–300	152.93	374.41
4	70.3	1500	–300	154.50	372.99
5	75	2000	–300	158.00	352.12
6	75	1500	–150	153.78	348.77
7	70.3	1000	–150	156.04	373.60
8	65	500	–150	151.19	384.33
9	60	100	–150	141.98	369.31
10	55	2000	–150	153.59	368.00
11	55	1500	0	153.54	375.03
12	60	1000	0	151.61	374.77
13	65	2000	0	150.83	357.68
14	70.3	100	0	149.91	378.05
15	75	500	0	153.95	352.47
16	75	100	150	149.86	337.76
17	70.3	500	150	146.91	359.81
18	65	1500	150	149.37	360.83
19	60	2000	150	151.92	361.22
20	55	1000	150	149.53	367.74
21	55	500	300	140.64	379.01
22	60	1500	300	150.07	357.97
23	65	100	300	146.81	380.19
24	70.3	2000	300	149.61	338.71
25	75	1000	300	149.65	328.52

**Table 3:** Range analysis of hardness and Young’s modulus.

Factor	Indenter angle θ (°)		Tip radius *r* (nm)		Residual stress *σ* (MPa)	
Mechanical properties	HIT	EIT	HIT	EIT	HIT	EIT
$\bar{K1}$	375.19	147.97	358.00	144.91	361.35	150.45
$\bar{K2}$	369.16	149.29	371.63	148.70	368.80	151.32
$\bar{K3}$	371.49	150.23	363.80	151.95	367.60	151.97
$\bar{K4}$	364.63	151.39	363.12	152.25	357.47	149.52
$\bar{K5}$	343.93	153.05	355.55	152.79	356.88	147.36
*R*_1_	31.26		16.08		11.92	
*R*_2_		5.08		7.88		4.61

Variables *R*_*θ*_, *R*_*r*_, *R*_*σ*_ represent the ranges in indenter angle, tip radius, and residual stress, respectively. For the ranges of hardness in Table 3, *R*_*θ*1_ = 31.26, *R*_*r*1_ = 16.08, and *R*_*σ*1_ = 11.92. Similarly, the ranges of Young’s modulus are *R*_*θ*2_ = 5.08, *R*_*r*2_ = 7.88, and *R*_*σ*2_ = 4.61. Thus, it can be seen that *R*_*θ*_, *R*_*r*_>*R*_*σ*_, which shows that the influences of angle and tip radius on the mechanical properties are more remarkable than that of residual stress. In other words, the influence of experimental error is greater than the effect of residual stress. It is mainly caused by the change of internal shear stress between indenter and specimen. Therefore, it is imperative to select an accurate indenter before conducting nanoindentation. Meanwhile, for three factors, *R*_1_>*R*_2_, indicated the sensitivity of hardness to the indenter shape is higher than that of elasticity. The Young’s modulus is an intrinsic material parameter and should not vary unless the material's size effects are taken into account in the constitutive law used in the simulation [27]. Hence, the influence of indenter shape is mainly reflected in hardness measurement.

### Estimation of the mechanical properties of materials

**Fig 8:**
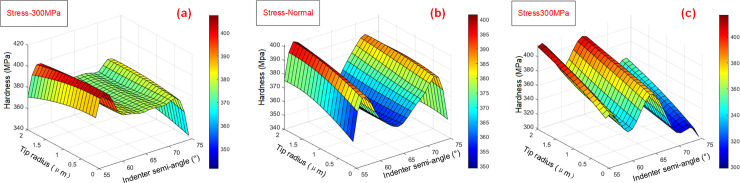
Effects of indenter shape on measured hardness under different residual stresses. (a) residual stress at –300 MPa, (b) no stress state, (c) residual stress at 300 MPa.

Hardness was chosen as the evaluation index. The influences of indenter angle and tip radius on measured hardness under different residual stresses are shown in Fig 8. It shows that with increases in tip radius, the overall hardness value presents a linear trend. However, the changes of indenter angle will affect a large deviation and fluctuation of the measurement results. The pile-up height of the workpiece surface will be influenced by the loading force at the fixed depth used in the nanoindentation experiment, which is the main cause of the error. When the residual stress is −300 MPa, hardness measurement results of the indenter with a 55° conical half-angle and 100 nm tip radius is 9.67% greater than that of an indenter with a 75° conical half-angle and 2000 nm tip radius.

By combining Figs 7 and 8, it can be seen that the influences of residual stress on hardness and elasticity modulus are approximately linear. With changes in residual stress, the influences of indenter angle and tip radius on mechanical performance vary. As the indenter angle changes from 50° to 70°, the overall hardness curve is wavy and the hardness changes linearly with increases in tip radius. Considering the interaction between the dependent variables, a multivariate nonlinear model was designed, as per Eq (11):

$$\hat{y}=b_{0}+b_{1}{x_{1}}^{3}+b_{2}{x_{1}}^{2}+b_{3}x_{1}+b_{4}x_{2}+b_{5}x_{3}+b_{6}x_{1}x_{3}+b_{7}x_{2}x_{3}$$ (11)

Where $\hat{y}$ represents the measured hardness of the material, *x*_1_, *x*_2_, *x*_3_ represent the indenter angle, tip radius, and residual stress respectively. Through a regression analysis of the orthogonal experimental data, *b*_0_ = 4224.747, *b*_1_−0.016, *b*_2_ = 2.961, *b*_3_ = −185.025, *b*_5_ = 0.155, *b*_6_ = −0.003, and *b*_4_ = *b*_7_ = 0. After fitting, it is proved that the angle change has a more significant impact on the hardness measurement results. The effect of tip radius on hardness can be ignored, the predictive formula for hardness is:

$$H_{it}=4224.747-0.016\theta^{3}+2.961\theta^{2}-185.025\theta +0.155\sigma^{x}-0.003\sigma^{x}\theta$$ (12)

where *σ*^*x*^ is the uniaxial residual stress in the material (MPa), *θ* is the conical half-angle of the indenter (°), *R* is the tip radius (nm), and *H*_*it*_ is the hardness at an indentation depth of 300 nm under various residual stresses (MPa).

A linear regression model was fitted to the observed values, with the determination coefficient *R*^2^ used to measure the goodness of fit [28]. The *R*^*2*^ value can range from 0 to 1, which higher values indicating a better fit. The expression is as follows:

$$R^{2}=\frac{SSR}{SST}=1-\frac{SSE}{SST}$$ (13)

Where *SST* is the total sum of squares, *SSR* is the sum of regression squares, and *SSE* is the sum of residual squares. The calculated *R*^2^ value for Eq (12) is 0.910, indicating that the predicted hardness values are in good agreement with the measured values.

**Fig 9:**
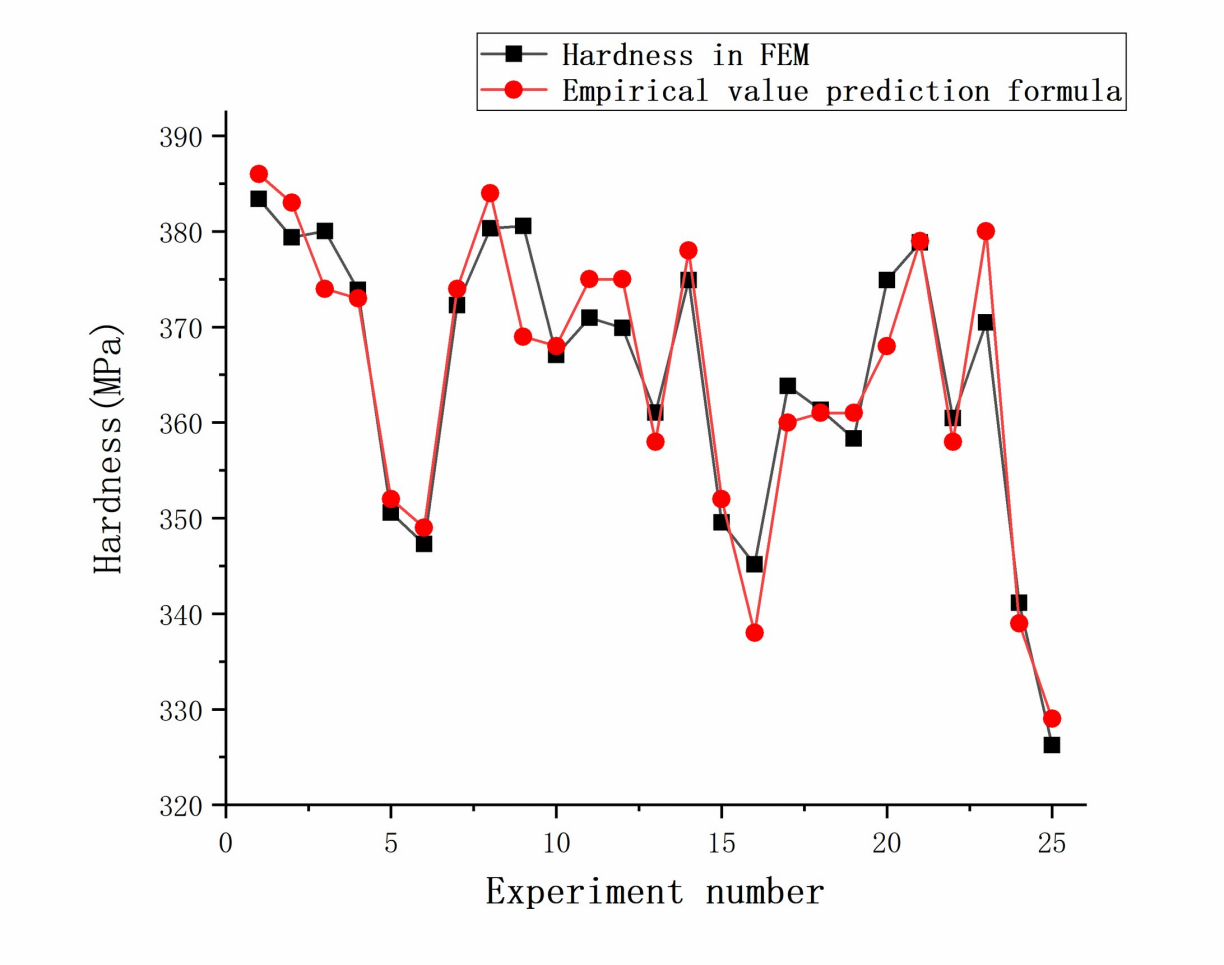
Fitting degree of the empirical formula.

Different indexes of indenter angle, tip radius, and measured hardness were brought into the empirical formula to obtain predicted values. The predicted values were compared with the hardness values in the orthogonal experimental table. Except for groups 9 and 23, the fitting accuracy of other groups was high and the hardness errors ranged between 0.04% and 3.14%, which may be due to the accuracy of the finite element model and mesh, as shown in Fig 9. Considering these errors, the mechanical properties of Inconel 718 estimated with the proposed formulas at an indentation depth of 300 nm provide some useful information.

## Conclusions

Based on a gradient material model, FEM and orthogonal experiments were used to study the effect of residual stress on the nanoindentation results of the machined subsurface of Inconel 718 alloy. Evidence shows that gradient materials' mechanical properties are mainly reflected on the top, and different stress states will affect the pile-up height of the material surface after nanoindentation. This leads to inaccurate measurements. Meanwhile, indenter wear and changes in geometric angle and tip radius can aggravate the measurement error. A three-factor and five-level orthogonal experiment considered the geometric shape of the indenter and residual stress. Results reveal that the indenter angle is the greatest impact factor on the measurement results. Then, an empirical formula was obtained for predicting the effect of internal uniaxial residual stress on the nanoindentation results.

1. Gradient materials and surface piling-up are not considered in the traditional nanoindentation test method, which results in less accurate results. When the uniaxial residual stress changes from −300 MPa to 300 MPa, the hardness and Young's modulus decrease by about 37 MPa and 6.6 GPa, respectively. By introducing a model of the mechanical properties under the influence of residual stress, the influence of residual stress on the subsurface of Inconel 718 can be predicted.
2. Through the analysis of orthogonal tests of residual stress, indenter angle, and tip radius, it was found that the sensitivity of mechanical property test results to residual stress is the lowest. For hardness measurements, the influence of the indenter angle is more significant than that of tip radius.
3. Hardness measurement values were characterized by three different indexes—residual stress, indenter angle, and tip radius—and a numerical model was established. It was found that the hardness error was 0.04–3.14%, which indicates good reliability. Therefore, in practical applications, this model can be used to predict the influence of residual stress at measurement depths of 0–300 nm.

## Supporting information

S1 File Fitting result. (DOCX)
