# Supplementary material for: Measuring the effect of residual stress on the machined subsurface of Inconel 718 by nanoindentation
Source: PLoS One. 2021 Jan 14;16(1):e0245391. doi: 10.1371/journal.pone.0245391 (PMC7808621; doi:10.1371/journal.pone.0245391)
Supplement: S1 File — (DOCX) [file pone.0245391.s001.docx]

S1 File.

**Nonlinear regression analysis（Data from SPSS）**

| **Iteration history^b^** | | | | | | | |
| --- | --- | --- | --- | --- | --- | --- | --- |
| Number of iterations |  | parameter | | | | | |
|  | Residual sum of squares | b0 | b1 | b2 | b3 | b4 | b5 |
| 1.0 | 3334098.640 | .000 | .000 | .000 | .000 | .000 | .000 |
| 1.1 | 2496445.225 | 9.656 | 3.021E-5 | .002 | .146 | .006 | .000 |
| 2.0 | 2496445.225 | 9.656 | 3.021E-5 | .002 | .146 | .006 | .000 |
| 2.1 | 1209297.821 | 29.516 | 9.064E-5 | .006 | .443 | .019 | -.003 |
| 3.0 | 1209297.821 | 29.516 | 9.064E-5 | .006 | .443 | .019 | -.003 |
| 3.1 | 131632.781 | 87.890 | .000 | .015 | 1.181 | .028 | -.014 |
| 4.0 | 131632.781 | 87.890 | .000 | .015 | 1.181 | .028 | -.014 |
| 4.1 | 10792.430 | 223.587 | .000 | .010 | 2.040 | .028 | .001 |
| 5.0 | 10792.430 | 223.587 | .000 | .010 | 2.040 | .028 | .001 |
| 5.1 | 759.341 | 239.491 | .000 | .074 | 1.394 | .000 | .155 |
| 6.0 | 759.341 | 239.491 | .000 | .074 | 1.394 | .000 | .155 |
| 6.1 | 739.304 | 401.396 | -.002 | .194 | -6.251 | .000 | .155 |
| 7.0 | 739.304 | 401.396 | -.002 | .194 | -6.251 | .000 | .155 |
| 7.1 | 704.702 | 704.034 | -.003 | .413 | -20.402 | .000 | .155 |
| 8.0 | 704.702 | 704.034 | -.003 | .413 | -20.402 | .000 | .155 |
| 8.1 | 644.054 | 1309.348 | -.005 | .851 | -48.706 | .000 | .155 |
| 9.0 | 644.054 | 1309.348 | -.005 | .851 | -48.706 | .000 | .155 |
| 9.1 | 556.982 | 2519.975 | -.009 | 1.727 | -105.313 | .000 | .155 |
| 10.0 | 556.982 | 2519.975 | -.009 | 1.727 | -105.313 | .000 | .155 |
| 10.1 | 511.741 | 4224.753 | -.016 | 2.961 | -185.025 | .000 | .155 |
| 11.0 | 511.741 | 4224.753 | -.016 | 2.961 | -185.025 | .000 | .155 |
| 11.1 | 511.741 | 4224.747 | -.016 | 2.961 | -185.025 | .000 | .155 |

| **Iteration history^b^** | | |
| --- | --- | --- |
| Number of iterations | parameter | |
|  | b6 | b7 |
| 1.0 | .000 | .000 |
| 1.1 | -1.278E-5 | 9.613E-5 |
| 2.0 | -1.278E-5 | 9.613E-5 |
| 2.1 | -4.055E-5 | .000 |
| 3.0 | -4.055E-5 | .000 |
| 3.1 | .000 | .000 |
| 4.0 | .000 | .000 |
| 4.1 | .000 | .000 |
| 5.0 | .000 | .000 |
| 5.1 | -.003 | .000 |
| 6.0 | -.003 | .000 |
| 6.1 | -.003 | .000 |
| 7.0 | -.003 | .000 |
| 7.1 | -.003 | .000 |
| 8.0 | -.003 | .000 |
| 8.1 | -.003 | .000 |
| 9.0 | -.003 | .000 |
| 9.1 | -.003 | .000 |
| 10.0 | -.003 | .000 |
| 10.1 | -.003 | .000 |
| 11.0 | -.003 | .000 |
| 11.1 | -.003 | .000 |

| **Parameter estimate** | | | | |
| --- | --- | --- | --- | --- |
| parameter |  | | 95% confidence interval | |
|  | estimation | standard error | Lower limit | Upper limit |
| b0 | 4224.747 | 1391.828 | 1288.247 | 7161.247 |
| b1 | -.016 | .005 | -.027 | -.005 |
| b2 | 2.961 | 1.007 | .837 | 5.085 |
| b3 | -185.025 | 65.028 | -322.223 | -47.827 |
| b4 | .000 | .016 | -.034 | .032 |
| b5 | .155 | .051 | .049 | .262 |
| b6 | -.003 | .001 | -.005 | -.001 |
| b7 | .000 | .000 | .000 | .000 |

| **Correlation of parameter estimates** | | | | | | | | |
| --- | --- | --- | --- | --- | --- | --- | --- | --- |
|  | b0 | b1 | b2 | b3 | b4 | b5 | b6 | b7 |
| b0 | 1.000 | -.997 | .998 | -1.000 | -.012 | -.002 | .002 | .011 |
| b1 | -.997 | 1.000 | -1.000 | .999 | .000 | .000 | .000 | .000 |
| b2 | .998 | -1.000 | 1.000 | -1.000 | .000 | .000 | .000 | .000 |
| b3 | -1.000 | .999 | -1.000 | 1.000 | .004 | .001 | .000 | -.004 |
| b4 | -.012 | .000 | .000 | .004 | 1.000 | .155 | -.186 | -.994 |
| b5 | -.002 | .000 | .000 | .001 | .155 | 1.000 | -.994 | -.186 |
| b6 | .002 | .000 | .000 | .000 | -.186 | -.994 | 1.000 | .217 |
| b7 | .011 | .000 | .000 | -.004 | -.994 | -.186 | .217 | 1.000 |

| **ANOVA^a^** | | | |
| --- | --- | --- | --- |
| Root | sum of square | df | mean square |
| regression | 3333586.899 | 8 | 416698.362 |
| Residual | 511.741 | 17 | 30.102 |
| Uncorrected total | 3334098.640 | 25 |  |
| Corrected total | 5694.549 | 24 |  |
| Dependent variable: HIT | | | |
| a. R^2^ = 1 -（SSE）/（SST）= .910。 | | | |
